# Methylene blue-photodynamic therapy for *Microsporum canis* infection: investigating a dual mechanism of fungicidal action and neutrophil homeostasis restoration

**DOI:** 10.3389/fmicb.2025.1668703

**Published:** 2025-11-17

**Authors:** Xinyu Zhang, Gaoyuan Peng, Kaisu Pan, Shulei Qin, Weilun Xu, Lan Huang, Liuwei Liao, Qian Lu, Qihua Huang, Shao Haotian, Dongyan Zheng, Cunwei Cao

**Affiliations:** 1 Department of Dermatology and Venereology, The First Affiliated Hospital of Guangxi Medical University, Nanning, China; 2 Guangxi Scientific and Technological Innovation Cooperation Base of Mycosis Prevention and Control, Nanning, China; 3 Guangxi Key Laboratory of Mycosis Prevention and Treatment, Nanning, China; 4 Guangxi Key Laboratory of AIDS Prevention and Treatment, Guangxi Medical University, Nanning, Guangxi, China; 5 Fangchenggang Wanqing Institute of Mycosis Prevention and Control, Fangchenggang, China

**Keywords:** *Microsporum canis*, photodynamic therapy, methylene blue, neutrophils, immunomodulation, tinea capitis

## Abstract

**Background:**

*Microsporum canis* is an increasingly common cause of tinea capitis. Conventional antifungal therapies are limited by toxicity and resistance, creating a need for novel treatments. Antimicrobial photodynamic therapy (aPDT) is a promising alternative. We investigated the efficacy and dual mechanism of methylene blue-photodynamic therapy (MB-PDT) against *M. canis*, focusing on its effects on the host innate immune response.

**Methods:**

The *in vitro* susceptibility of clinical *M. canis* isolates was determined by broth microdilution. Fungal ultrastructural changes were examined using transmission electron microscopy. *In vivo* efficacy was assessed in a murine dermatophytosis model and compared to topical terbinafine. Systemic immunomodulatory effects were evaluated by flow cytometric analysis of peripheral blood neutrophil phenotypes (Dectin-1, Dectin-2) and functional markers (MPO, NOX2).

**Results:**

*In vitro*, MB-PDT demonstrated potent fungicidal activity (Geometric Mean MIC at 80 J/cm^2^: 0.367 μg/mL; 95% CI: 0.295–0.439 μg/mL). It induced severe ultrastructural damage, including mitochondrial collapse and cell wall disruption. In the murine model, MB-PDT achieved an 80% mycological cure rate, significantly outperforming topical terbinafine (20% cure rate). Mechanistically, *M. canis* infection induced systemic neutrophil dysfunction, evidenced by a population shift and suppressed MPO and NOX2 expression. MB-PDT treatment reversed this immune dysfunction, restoring neutrophil homeostasis and the expression of key functional markers (MPO, NOX2).

**Conclusion:**

MB-PDT is a highly effective treatment for *M. canis* infection. Its efficacy is based on a dual mechanism: direct fungicidal action through oxidative damage and restoration of host neutrophil function.

## 1 Introduction

Dermatophytoses, superficial fungal infections of keratinized tissues, constitute a significant global health burden, affecting over 1.7 billion people (Chen et al., 2018; Brown et al., 2012). The epidemiology of these infections is dynamic, with recent data indicating a marked increase in the prevalence of the zoophilic fungus *Microsporum canis* as a primary agent of tinea capitis, particularly in children (Zhan and Liu, 2017; Rodríguez-Cerdeira et al., 2021; Zheng et al., 2023). This epidemiological trend is closely linked to the growing popularity of domestic pets, which serve as the main reservoir for *M. canis* transmission (Moriello et al., 2017; Zhan et al., 2015). If not managed effectively, tinea capitis can progress to severe inflammatory forms, such as Kerion, leading to permanent scarring alopecia and considerable psychological distress (John et al., 2018; Hay, 2017).

Standard management of tinea capitis involves prolonged courses of systemic antifungals, such as griseofulvin, terbinafine, or Iitraconazole (Mayser et al., 2020; Kakourou and Uksal, 2010). However, this approach presents significant clinical challenges, especially in pediatric patients. These include the burden of long-term treatment, risks of hepatotoxicity, and drug–drug interactions. Compounding these issues are the variable efficacy against *M. canis* and the growing global concern of antifungal resistance, which collectively underscore an urgent need for safer, more effective localized therapies (Pereira, 2021; Surur et al., 2024; Chen et al., 2016).

Antimicrobial photodynamic therapy (aPDT) has emerged as a promising non-antibiotic approach that directly addresses these limitations (Almenara-Blasco et al., 2024). As a topical modality, it avoids systemic side effects. This modality combines a non-toxic photosensitizer (PS), visible light of an appropriate wavelength, and ambient oxygen to generate cytotoxic reactive oxygen species (ROS), which induce rapid, multi-targeted microbial killing (Kwiatkowski et al., 2018; Cieplik et al., 2017). A pivotal advantage of this non-specific mechanism is the extremely low likelihood of inducing microbial resistance, a key advantage over conventional antifungals (Surur et al., 2024; Tiwari et al., 2025). Methylene blue (MB), a phenothiazinium dye, is a well-suited PS for dermatological use due to its established safety profile, low cost, and strong absorbance of red light (≈668 nm), which allows for the tissue penetration required to target pathogens within hair follicles (Lee et al., 2013; Wu and Hu, 2022). While the efficacy of MB-PDT has been demonstrated against fungi like *Candida albicans* and *Trichophyton* species (Chen et al., 2019; López-Chicón et al., 2016), its activity against *M. canis* is largely uncharacterized, with evidence limited to isolated veterinary case reports Cabral et al. (2021).

Successful pathogen clearance depends on the synergy between an antimicrobial agent and the host immune system, where neutrophils are the principal effector cells of the innate antifungal response (Borregaard, 2010). Neutrophils recognize fungal pathogens through pattern recognition receptors (PRRs), including the C-type lectin receptors Dectin-1 (recognizing β-glucans) and Dectin-2 (recognizing α-mannans) (Kerrigan and Brown, 2010; Nikolakopoulou et al., 2020). This recognition triggers potent effector functions, including the phagocytic oxidative burst mediated by NADPH oxidase 2 (NOX2) and the degranulation of enzymes like myeloperoxidase (MPO) (Taylor et al., 2007; Saijo et al., 2010). Although the direct microbicide effects of aPDT are well-established, and some studies have noted its influence on local cytokine profiles or macrophage activity, its systemic impact on the functional state of neutrophils—the primary effector cells in antifungal defense—during dermatophyte infection remains a critical and largely unexplored area of investigation.

This study was therefore designed as a proof-of-concept investigation to evaluate the *in vitro* and *in vivo* efficacy of MB-PDT against *M. canis* and to characterize its potential immunomodulatory effects. Accordingly, we hypothesized that the observed therapeutic effect is attributable to a dual mechanism. This proposed mechanism includes: (1) a direct fungicidal action resulting in significant ultrastructural damage to the pathogen, and (2) a modulation of the host’s innate immune response, indicated by a restoration of neutrophil homeostasis and function.

## 2 Materials and methods

### 2.1 Fungal strains and inoculum preparation

Twenty-four unique clinical isolates of *M. canis* were prospectively collected from pediatric patients (ages 3–12) presenting with clinically and mycological confirmed tinea capitis at the Dermatology Clinic of The First Affiliated Hospital of Guangxi Medical University (Nanning, China) between January 2019 and May 2022. For each isolate, species-level identification was rigorously confirmed using a dual approach: (1) classical morphological analysis, including macroscopic colony characteristics on Potato Dextrose Agar (PDA) and microscopic features (e.g., macroconidia morphology) after lacto phenol cotton blue staining, and (2) molecular identification via sequencing of the internal transcribed spacer (ITS) region of the ribosomal DNA. All 24 isolates showed >99% sequence homology to a certified *M. canis* reference strain in the Gen Bank database. For experimental use, isolates were cultured on Potato Dextrose Agar (PDA; Hopebio, Qingdao, China) at 28 °C for 10–14 days. Fungal inoculate were prepared following the guidelines of the Clinical and Laboratory Standards Institute (CLSI) document M38-A3 (Wayne and Clinical and Laboratory Standards Institute (CLSI), 2017). Conidia and hyphal fragments were harvested by gently scraping mature cultures in sterile saline with 0.05% Tween 80. The suspension was adjusted using a hemocytometer to a final concentration of 2–3 × 10^5^ CFU/mL for *in vitro* assays or 1 × 10^9^ CFU/mL for the *in vivo* infection model.

### 2.2 Photosensitizer and light source

Clinical-grade Methylene Blue (MB; Macklin, Shanghai, China) was dissolved in deionized water to create a 1% (w/v) stock solution, which was stored protected from light. Irradiation was performed using a red-light LED device (PL-D, Peninsula Medical, Chongqing, China) with a peak emission wavelength of 632 ± 10 nm. The light flounce (J/cm^2^) was controlled by adjusting exposure time and monitored with a calibrated optical power meter.

### 2.3 *In vitro* antifungal photodynamic susceptibility testing

A broth microdilution assay was conducted in 96-well microtiter plates. Two-fold serial dilutions of MB (final concentrations 0.09375 to 48 μg/mL) were prepared in RPMI-1640 medium buffered with 3-(N-morpholino) propanesulfonic acid (MOPS). Each well was inoculated with 100 μL of the standardized *M. canis* suspension. Following a 20-min dark incubation at room temperature to facilitate PS uptake, plates were irradiated with red light at flounces ranging from 60 to 150 J/cm^2^. Control groups included: (i) MB without light (dark toxicity); (ii) light without MB (photo toxicity); and (iii) fungi alone (growth control). Plates were incubated at 28 °C for 72 h. The Minimum Inhibitory Concentration (MIC) was determined visually as the lowest MB concentration showing no visible growth.

### 2.4 Ultrastructural analysis by transmission electron microscopy (TEM)

*Microsporum canis* was treated with various concentrations of MB (0.1875, 0.375, 0.75, and 1.5 μg/mL) followed by irradiation (100 J/cm^2^), with an untreated culture serving as a control. Fungal pellets were fixed in 2.5% glutaraldehyde, post-fixed with 1% osmium tetroxide, dehydrated in a graded ethanol series, and embedded in EPON 812 resin. Ultrathin sections (60–80 nm) were stained with uranyl acetate and lead citrate and examined using a Hitachi HT7800 Transmission Electron Microscope (Hitachi, Tokyo, Japan).

### 2.5 Cytotoxicity assay

The cytotoxicity of MB was assessed on primary human dermal fibroblasts (HDF-a; ScienCell) and the immortalized human keratinocyte cell line HaCaT using the Cell Counting Kit-8 (CCK-8; Dojindo) assay. Cells were seeded in 96-well plates at a density of 3,000 cells/well and cultured for 24 h to allow for complete adherence (Mosmann, 1983; Guan et al., 2009). The experiment was designed with three distinct groups for each cell line: (i) a blank control group, containing only culture medium to correct for background absorbance; (ii) an untreated control group, containing cells in culture medium, which represented 100% viability; and (iii) experimental groups, containing cells treated with serial dilutions of MB (with concentrations ranging from 48 μg/mL down to 0.09375 μg/mL). After a 2.5-h incubation with MB in the dark, 10 μL of CCK-8 solution was added to each well, followed by another 2.5-h incubation at 37 °C. The absorbance was then measured at 450 nm using a microplate reader. Cell viability was calculated using the standard formula: Cell Viability (%) = [(A_sample − A_blank)/(A_untreated − A_blank)] × 100%. The half-maximal inhibitory concentration (IC50) was subsequently determined from the generated dose–response curves. All experiments were performed in triplicate and were repeated on three independent occasions.

### 2.6 Animal model of *Microsporum canis* dermatophytosis

All animal experiments were approved by the Institutional Animal Care and Use Committee of Guangxi Medical University (Approval No. 202303011). Forty male Kunming mice (8 weeks old, 35–40 g) were housed under specific pathogen-free conditions. Following established protocols for dermatophyte infection (Jin et al., 2009), a 3 × 5 cm area on the dorsum of each mouse was shaved and treated with a depilatory cream. The stratum corneum was gently disrupted using a 0.5 mm microneedle roller. *M. canis*. To establish the infection model, a representative clinical isolate (GXMU-MC-07) was selected from our collection of 24 strains. This isolate was chosen based on its robust growth characteristics, consistent sporulation, and typical virulence profile observed in preliminary screenings. A 200 μL aliquot of the concentrated inoculum of this isolate (1 × 10^9^ CFU/mL) was applied and occluded for 24 h. The inoculation was repeated on the following day to establish a robust infection.

### 2.7 *In vivo* treatment protocols

On day 7 post-infection, when clinical signs were evident, the mice were randomly allocated into four groups (*N* = 10 per group). This randomization was performed using a computer-based method to ensure complete impartiality. Specifically, each of the 40 mice was assigned a unique identification number (1–40). We then utilized the RAND() function in Microsoft Excel to generate a unique random number for each mouse. Subsequently, the entire list of mice was sorted in ascending order based on these generated random numbers. Finally, the first 10 mice on the sorted list were assigned to the MB-PDT group, the next 10 to the Terbinafine group, and so on, until all 40 mice were allocated to one of the four groups. This rigorous procedure ensured that every mouse had an equal probability of being assigned to any group, thereby eliminating potential allocation bias.

**MB-PDT Group:** A 0.1% (w/v) MB hydrogel was applied topically to the lesion and a 1 cm margin. After a 2-h dark incubation under occlusion, the gel was removed, and the area was irradiated with red light (100 J/cm^2^). Treatment was administered on days 7 and 10 post-infection.

**Terbinafine Group (Positive Control):** 1% terbinafine cream was applied daily from day 7 to day 13.

**Light-Only Group (Control):** Mice received a drug-free hydrogel and identical irradiation on days 7 and 10.

**Untreated Group (Control):** No treatment was given.

### 2.8 Evaluation of *in vivo* therapeutic efficacy

Efficacy was assessed on days 7, 11, and 14.

**Clinical Assessment:** Lesions were scored for erythema, scaling, and crusting on a 0–3 scale for each parameter (total score 0–9). The assessment was performed by two independent investigators who were blinded to the treatment group assignments to ensure objectivity.

**Mycological Assessment:** Hair and skin scrapings were collected for direct microscopy (20% KOH) and culture on PDA. Mycological cure was defined as negative findings in both tests, a method consistent with established mycological analyses for evaluating therapeutic efficacy (Majima et al., 2005).

**Histopathological Analysis:** On day 14, skin biopsies were fixed in 10% formalin, paraffin-embedded, and sectioned for Hematoxylin and Eosin (H&E) and Periodic acid-Schiff (PAS) staining.

### 2.9 Host immune response analysis

At 2, 3, and 5 weeks post-infection, peripheral blood was collected via the retro-orbital plexus. Neutrophils were isolated using an EasySep™ Mouse Neutrophil Enrichment Kit (Stem Cell Technologies, Vancouver, BC, Canada). To prevent non-specific antibody binding, cells were pre-incubated with TruStain FcX™ (anti-mouse CD16/32, Bio Legend). Cells were then stained with antibodies against CD45, CD11b, Ly-6G, Dectin-1, and Dectin-2. Following fixation and permeabilization, intracellular staining was performed for MPO and NOX2. Data were acquired on a Cytoflex S flow cytometer (Beckman Coulter, Brea, CA, USA).

### 2.10 Statistical analysis

Statistical analysis was performed using SPSS Statistics (Version 25.0, IBM Corp., Armonk, NY, USA). Differences between groups were assessed using one-way ANOVA with Tukey’s post-hoc test. Mycological cure rates were compared using Fisher’s exact test. A *p*-value < 0.05 was considered statistically significant. Unless otherwise specified, quantitative data are presented as mean ± standard deviation (SD), and proportional data are presented with 95% confidence intervals (CI).

## 3 Results

### 3.1 MB-PDT demonstrates potent and consistent *in vitro* activity against *Microsporum canis*

**Figure 1:**
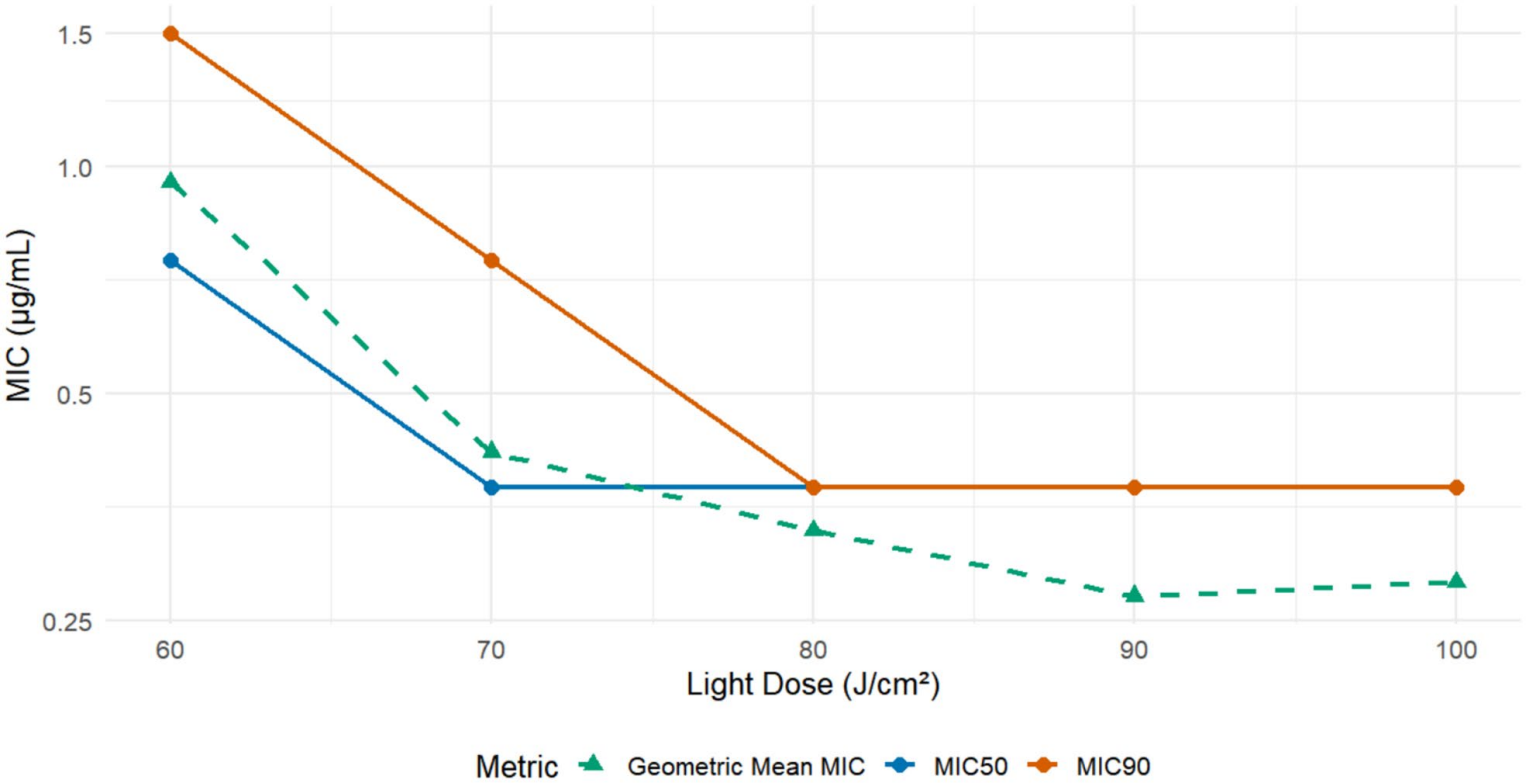
*In vitro* susceptibility of *M. canis* to Methylene Blue-Mediated Photodynamic Therapy (MB-PDT). The graph displays the *in vitro* antifungal activity of MB-PDT against 24 clinical isolates of *M. canis* at various light energy densities. The lines represent the Minimum Inhibitory Concentration required to inhibit 50% (MIC50) and 90% (MIC90) of isolates, as well as the Geometric Mean MIC. A clear light-dose-dependent increase in antifungal efficacy is observed, with activity plateauing at a light dose of 80 J/cm^2^, where the MIC90 was 0.375 μg/mL.

MB-PDT demonstrated potent, light- and concentration-dependent fungicidal activity against all 24 clinical *M. canis* isolates tested. No growth inhibition was observed in the dark toxicity (MB only) or phototoxicity (light only) control groups. As summarized in Figure 1, therapeutic efficacy increased with the applied light flounce, reaching a plateau at 80 J/cm^2^. At an energy density of 80 J/cm^2^, the geometric mean MIC was 0.367 μg/mL (95% CI: 0.295–0.439 μg/mL), with MIC₅₀ and MIC₉₀ values of 0.375 μg/mL, indicating robust and reliable antifungal action.

### 3.2 MB-PDT induces severe ultrastructural damage in fungal cells

**Figure 2:**
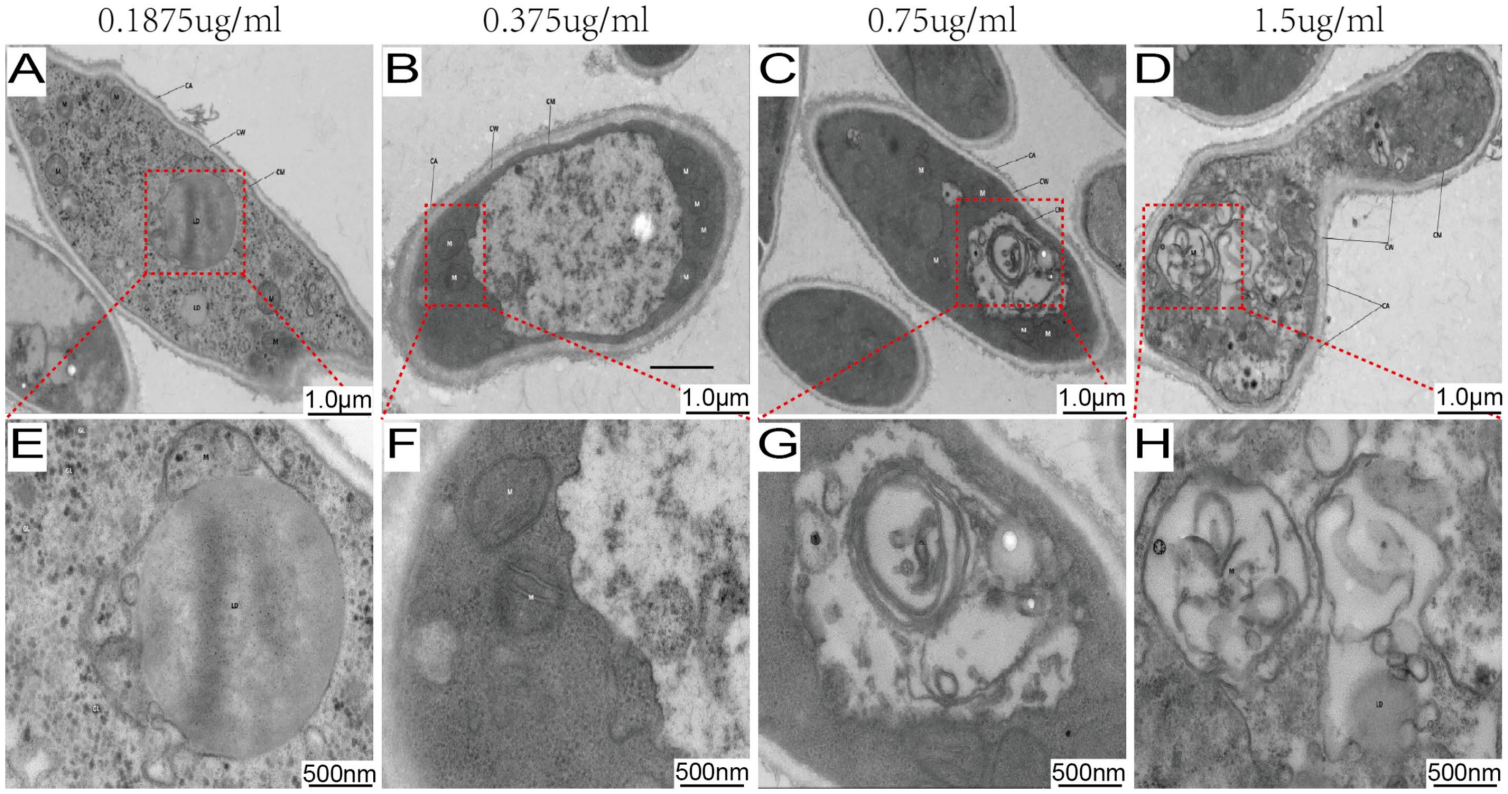
MB-PDT induces dose-dependent ultrastructural damage in *M. canis*. Transmission electron micrographs of *M. canis* after treatment with MB-PDT (100 J/cm^2^). **(A, E)** Treatment with 0.1875 μg/mL MB resulted in no obvious ultrastructural damage, showing an intact cell wall (CW), cell membrane (CM), and well-preserved organelles, including mitochondria (M) and a nucleus (N). **(B, F)** Treatment with 0.375 μg/mL MB, revealing early signs of damage such as mitochondrial swelling and slight cytoplasmic vacuolization. **(C, G)** Treatment with 0.75 μg/mL MB, showing severe damage with extensive vacuolization and mitochondrial collapse. **(D, H)** Treatment with 1.5 μg/mL MB, demonstrating severe destruction, including a compromised cell wall and complete disintegration of cytoplasmic contents. Scale bar = 500 nm.

Ultrastructural analysis by TEM revealed a dose-dependent gradient of cellular alterations (Figure 2). At the lowest concentration tested (0.1875 μg/mL), fungal cells showed no obvious signs of damage, remaining structurally intact with well-preserved organelles (Figure 2A). As the MB concentration increased, a progression of ultrastructural changes was observed. At 0.375 μg/mL, these changes included mitochondrial swelling and cytoplasmic vacuolization (Figure 2B). At higher concentrations (0.75–1.5 μg/mL), the alterations progressed to include mitochondrial collapse, cytoplasmic vacuolization, and compromised cell wall integrity (Figures 2C,D).

### 3.3 Favorable safety profile of methylene blue on human skin cells

**Figure 3:**
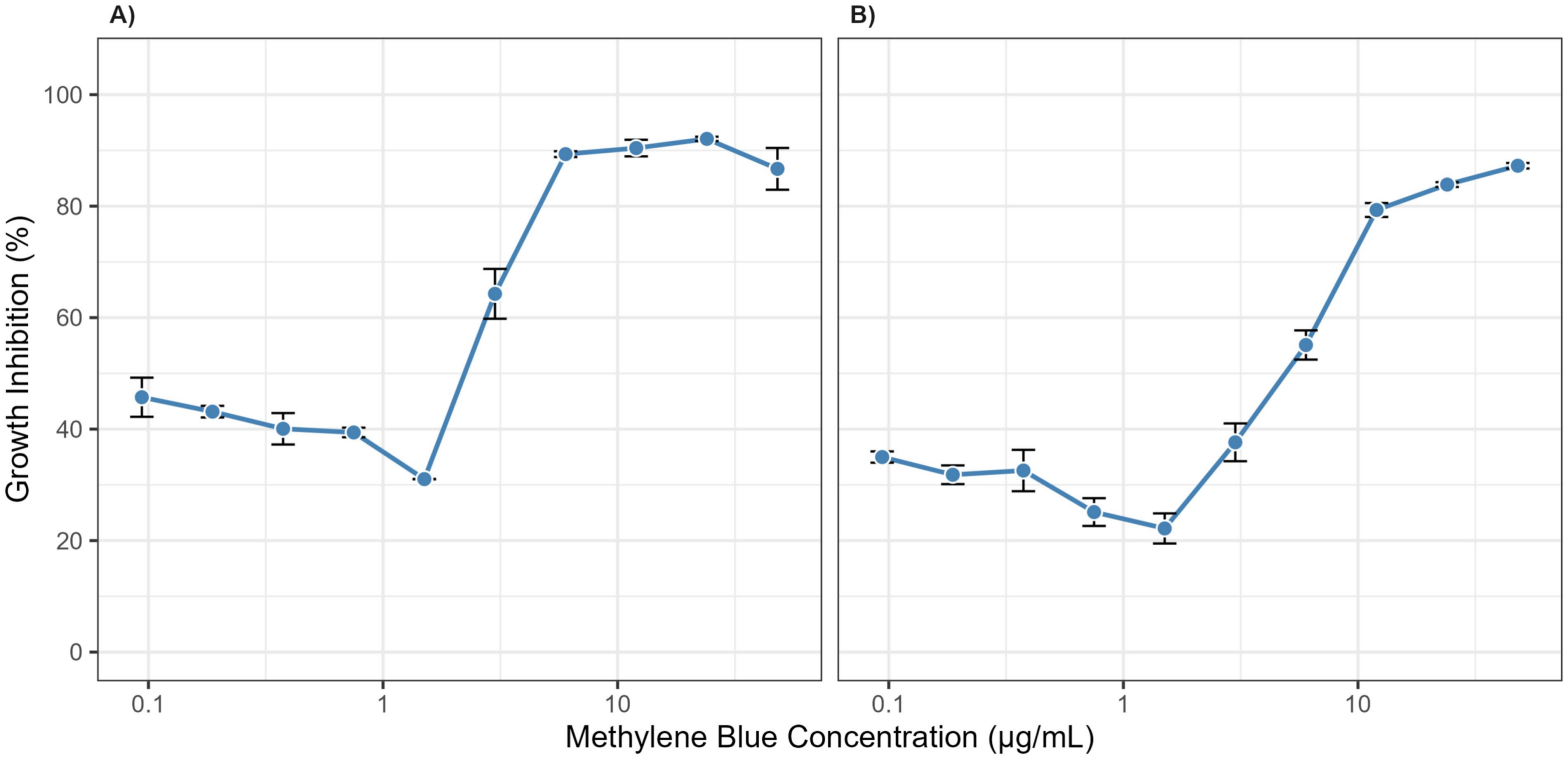
Cytotoxicity of methylene blue on human skin cells. Dose–response curves show the growth inhibition of **(A)** Human Dermal Fibroblasts (HDF-a) and **(B)** Human Keratinocytes (HaCaT) after 2.5 h of exposure to various concentrations of methylene blue. Data points represent the mean of three independent experiments, and error bars indicate the standard deviation (SD).

The cytotoxicity of MB was evaluated on HDF-a and HaCaT cells. As shown in Figure 3, MB displayed a favorable safety profile, with dose–response curves demonstrating low variability across replicates. The calculated IC50 values were 3.027 μg/mL for HDF-a and 0.7014 μg/mL for HaCaT. These concentrations are substantially higher than the effective antifungal MIC (≈0.3 μg/mL), indicating a significant therapeutic window with minimal expected toxicity to host cells.

### 3.4 Establishment and characterization of a clinically relevant murine infection model

**Figure 4:**
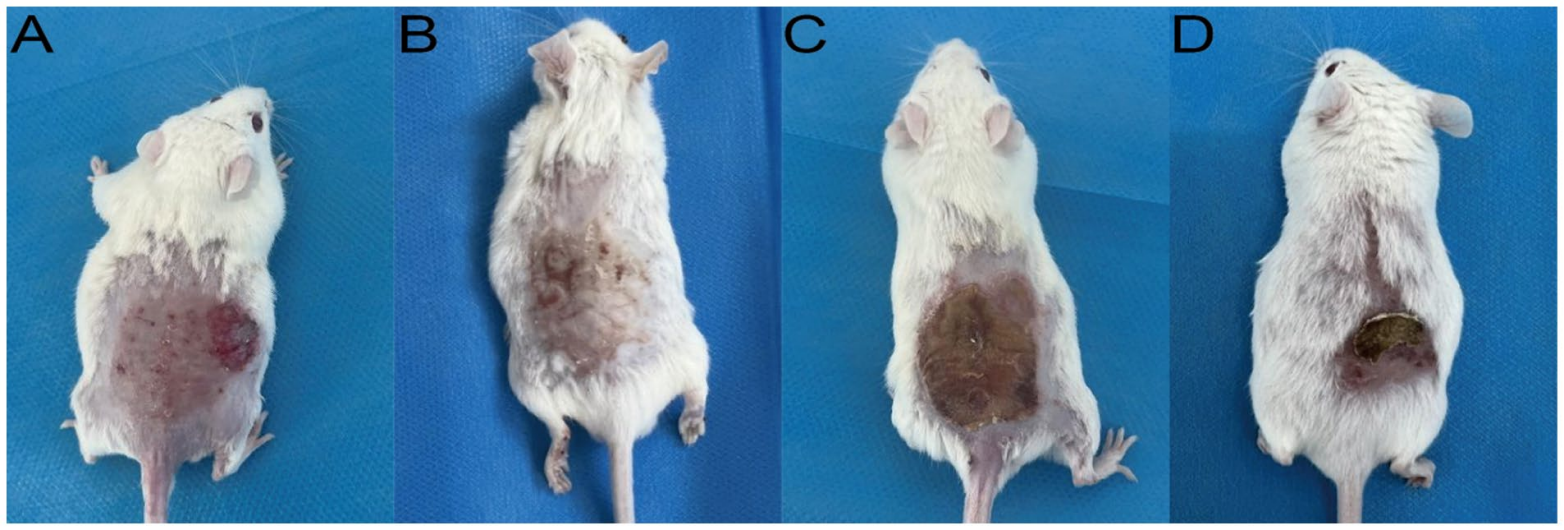
Clinical appearance of established *M. canis* infection in the murine model (Day 7). Representative images show **(A)** erythema and papules, **(B)** scaling, **(C)** adherent crusts, and **(D)** associated alopecia.

**Figure 5:**
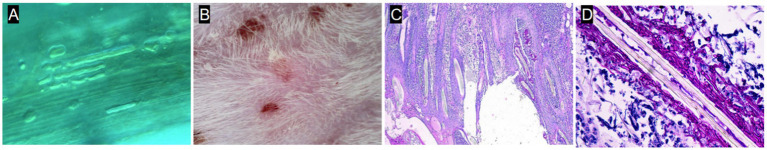
Mycological and histopathological characterization of the infection model. **(A)** Direct microscopy (KOH, 40×) showing endothrix infection. **(B)** Dermoscopy showing short villus hairs and scaling. **(C)** H&E stain showing a dense perifollicular inflammatory infiltrate. **(D)** PAS stain showing magenta-colored fungal elements within the hair follicle.

A robust and reproducible murine model of *M. canis* dermatophytosis was successfully established. By day 7 post-inoculation, all mice developed characteristic lesions, including erythema, scaling, and crusted alopecia (Figure 4). Mycological and histopathological analyses confirmed infection. Direct microscopy of plucked hairs revealed an endothrix pattern of infection (Figure 5A), while dermoscopy showed features of scaling and abnormal hair shafts (Figure 5B). Histopathology confirmed a dense, suppurative folliculitis (Figure 5C) with abundant PAS-positive fungal elements invading the hair follicles (Figure 5D).

### 3.5 Topical MB-PDT demonstrates superior therapeutic efficacy *in vivo*

**Figure 6:**
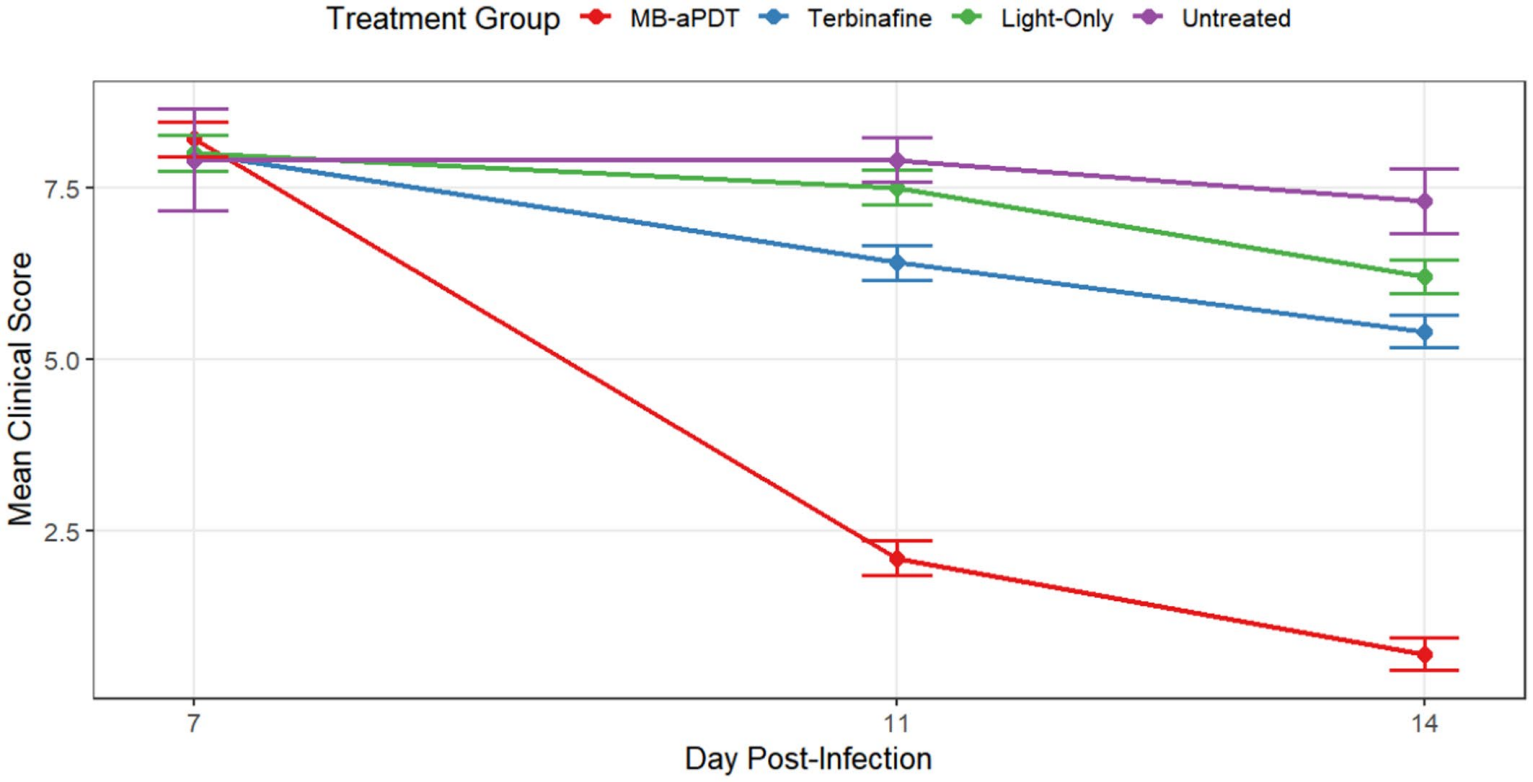
MB-PDT accelerates clinical recovery in a murine model of *M. canis* dermatophytosis. Comparison of mean clinical symptom scores over time in different treatment groups. Lesions were scored on days 7, 11, and 14 post-infection. The MB-PDT group demonstrated a rapid and statistically significant reduction in clinical scores compared to the terbinafine, light-only, and untreated control groups at both day 11 and day 14. Data are presented as mean ± SD (*N* = 10 mice per group). *p* < 0.001 for MB-PDT group compared to all other groups at the corresponding time point.

**Figure 7:**
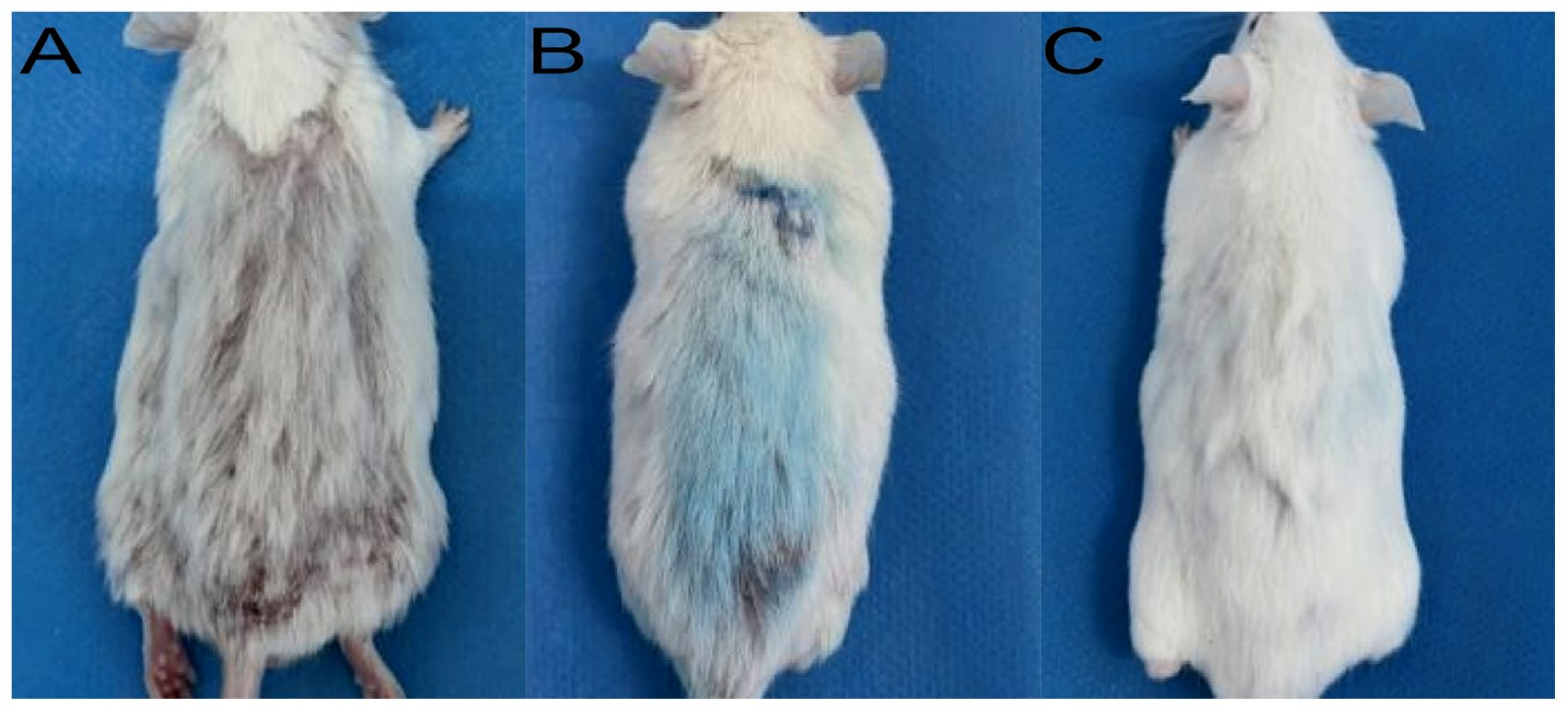
Clinical progression of healing in the MB-PDT treated group. Representative images showing a lesion at baseline **(A)**, after one treatment **(B)**, and after two treatments **(C)**, demonstrating rapid clinical resolution. This contrasts with the persistent lesion in a representative untreated control mouse at the study endpoint (Day 14; D).

**Figure 8:**
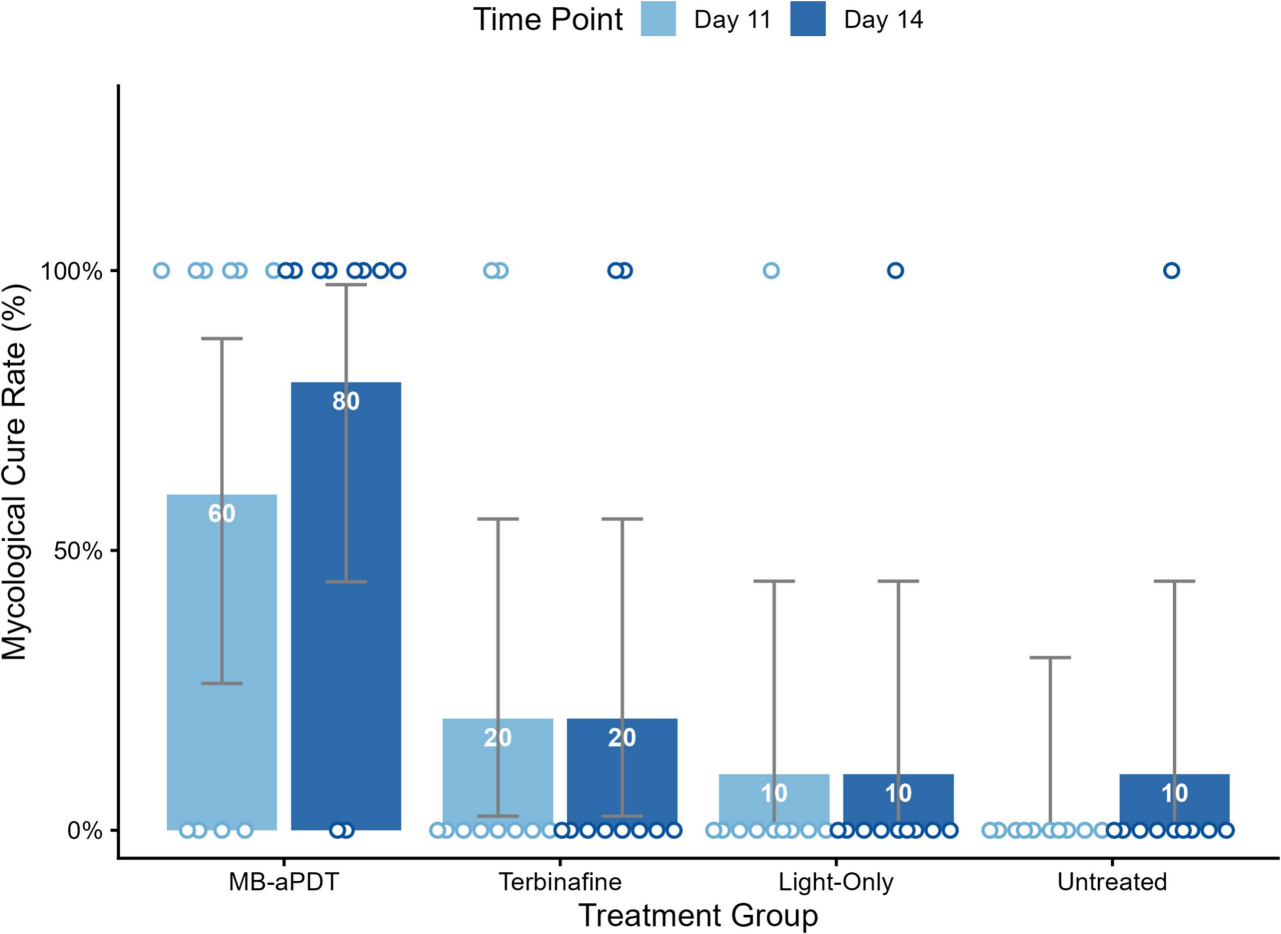
MB-PDT achieves superior mycological cure rates compared to conventional therapy. Bar chart augmented with individual data points, illustrating the mycological cure rates on day 11 and day 14 for all treatment groups. The bars represent the mean cure rate for each group, with the percentage value displayed inside. The superimposed circular points represent the outcome for each individual mouse (*N* = 10 per group), positioned at 0% for mycological failure (not cured) or 100% for mycological cure. The therapeutic superiority of MB-PDT was evident early; by day 11, its 60% cure rate was significantly higher than that of the terbinafine (20%), light-only (10%), and untreated (0%) groups. By day 14, the MB-PDT group achieved an 80% mycological cure rate, which remained significantly higher than the other groups. Error bars represent the 95% confidence interval (CI) for the proportion. *p* < 0.05 for the MB-PDT group compared to all other groups on both Day 11 and Day 14.

**Figure 9:**
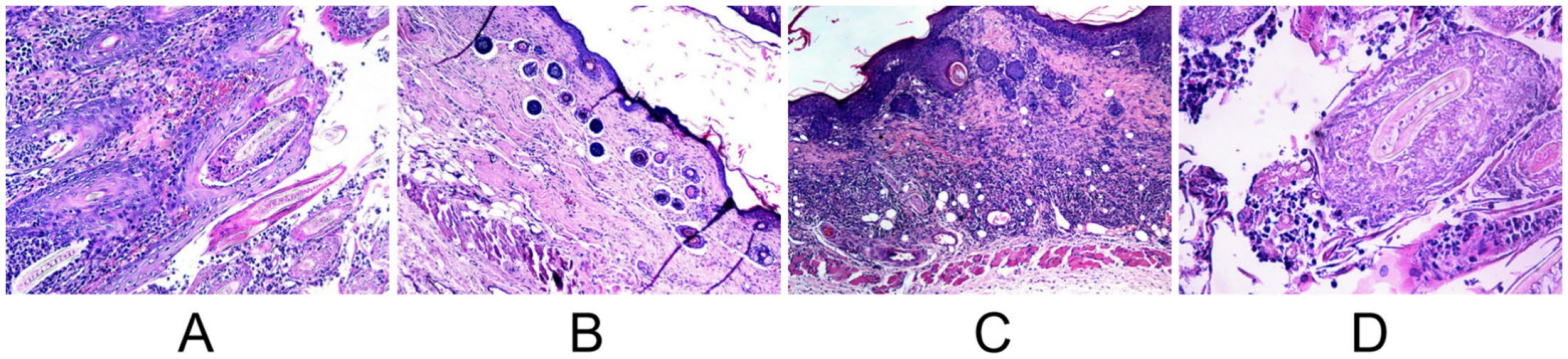
Comparative post-treatment histopathology (H&E stain) on day 14. **(A)** Untreated control showing persistent dense inflammation. **(B)** MB-PDT treated showing significant resolution of inflammation. **(C)** Terbinafine treated showing partial improvement. **(D)** Light-only control showing persistent inflammation.

In the murine infection model, MB-PDT demonstrated marked therapeutic superiority. The mean clinical lesion score in the MB-PDT group decreased from 8.2 ± 0.255 at baseline to 0.7 ± 0.244 on day 14, a significantly greater improvement than observed in the terbinafine, light-only, or untreated groups (*p* < 0.001) (Figures 6, 7). Critically, the therapeutic superiority of MB-PDT was evident at both time points assessed. Already by day 11, the MB-PDT group achieved a 60% mycological cure rate, which was significantly higher than the rates observed in the terbinafine (20%), light-only (10%), and untreated (0%) groups (*p* < 0.05 for all comparisons). This effect was further enhanced by day 14, where the cure rate in the MB-PDT group reached 80%, remaining significantly higher than the rates for topical terbinafine (20%) and control groups (10%) (*p* < 0.05) (Figure 8). The visualization in Figure 8 clearly illustrates this difference, showing that 8 of the 10 mice in the MB-PDT group achieved mycological cure, compared to only 2 of 10 in the terbinafine group and 1 of 10 in the control groups. This statistical significance is visually supported by both the clear separation in individual data points and the non-overlapping 95% confidence intervals. Histopathological analysis on day 14 confirmed the resolution of inflammation and clearance of fungal elements exclusively in the MB-PDT-treated group (Figure 9).

### 3.6 MB-PDT reverses pathological immune skewing and restores expression of neutrophil functional markers

**Figure 10:**
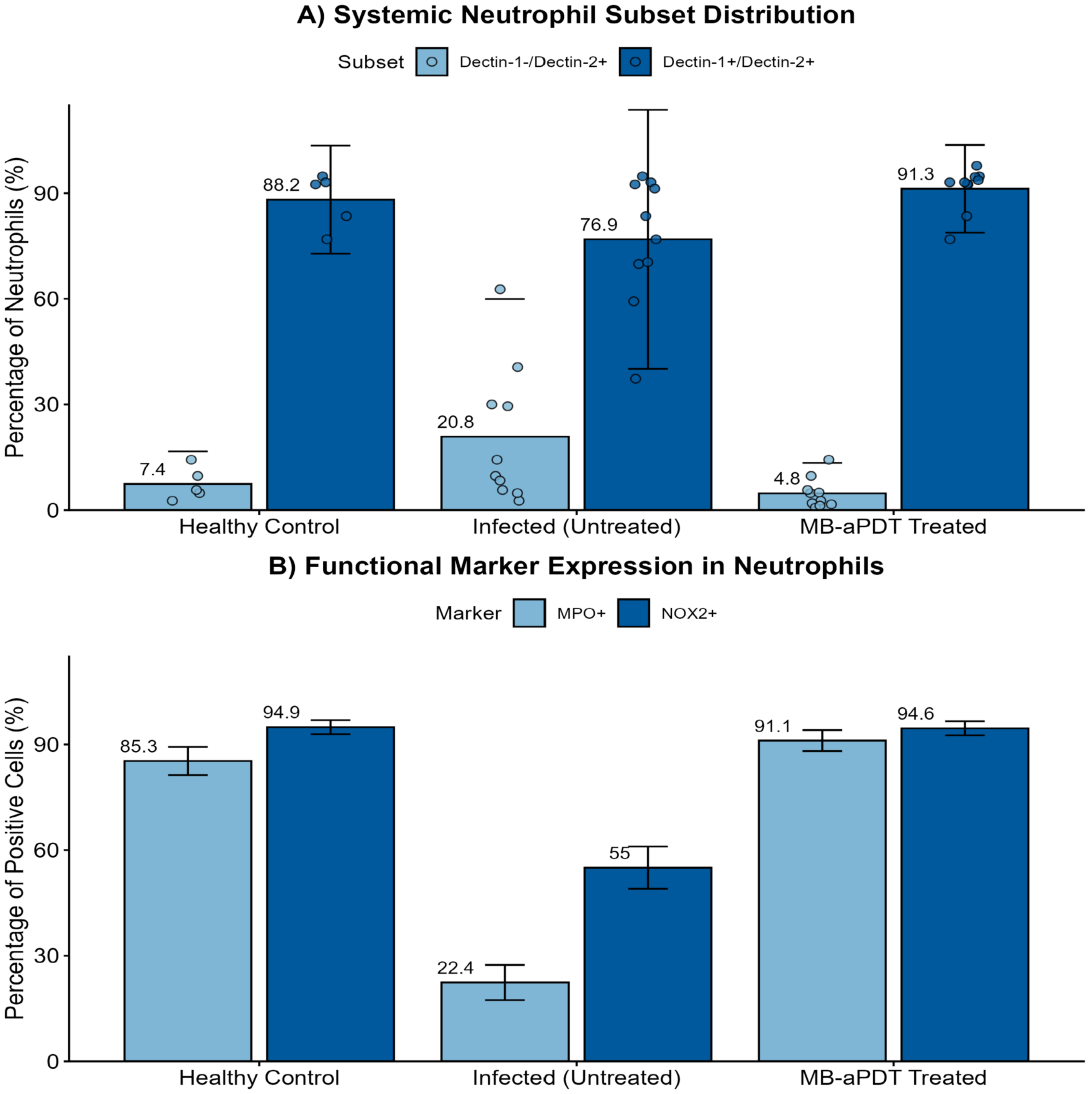
MB-PDT restores systemic neutrophil homeostasis and function during *M. canis* infection. Flow cytometry analysis of peripheral blood neutrophils from healthy control, infected (untreated), and MB-PDT treated mice. **(A)** *M. canis* infection induced a significant shift in neutrophil subsets, marked by an expansion of the inflammatory Dectin-1^−^/Dectin-2^+^ population compared to healthy controls (*p* < 0.05). MB-PDT treatment significantly reversed this dysregulation, restoring the homeostatic balance (*p* < 0.05 vs. infected group). **(B)** Similarly, infection led to a significant downregulation in the percentage of neutrophils expressing the key functional enzymes MPO and NOX2 (*p* < 0.05 for both vs. healthy controls). MB-PDT effectively restored the expression of both markers to levels significantly higher than those in the infected group (*p* < 0.05 for both). Data are presented as mean ± SD (*N* = 5 per group). In panel **(A)**, individual data points are shown to illustrate distribution. Error bars extending below the zero axis are truncated.

To investigate the immunomodulatory effects of the therapy, we analyzed peripheral blood neutrophils using flow cytometry. As shown in Figure 10, *M. canis* infection induced a profound shift in the systemic neutrophil profile. Compared to healthy controls, the proportion of the homeostatic Dectin-1+/Dectin-2+ subset was significantly reduced (from a mean of 88.2% in healthy controls to 65.6% in infected mice), while a distinct Dectin-1−/Dectin-2+ inflammatory subset expanded dramatically (from 7.4 to 34.2%) (Figure 10A). MB-PDT treatment effectively reversed this pathological shift, restoring the Dectin-1+/Dectin-2+ population to 94.4%. Furthermore, infection significantly reduced the percentage of neutrophils expressing the key antimicrobial enzymes MPO (from 85.3 to 22.4%) and NOX2 (from 94.9 to 55.0%). MB-PDT treatment also restored the expression of these functional markers to levels comparable with healthy controls (Figure 10B).

## 4 Discussion

**Figure 11:**
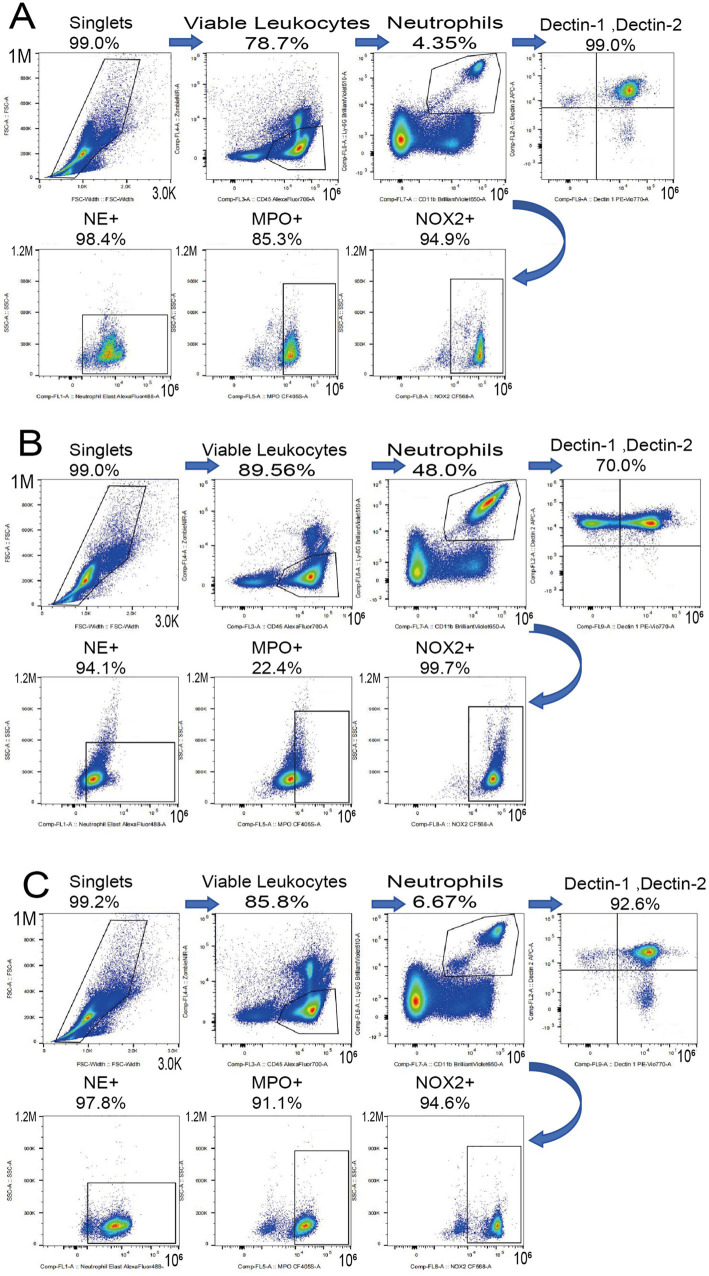
MB-PDT restores systemic neutrophil homeostasis. Representative flow cytometry plots showing Dectin-1 and Dectin-2 expression on peripheral blood neutrophils from **(A)** a healthy control, **(B)** an untreated infected mouse, and **(C)** an MB-PDT treated mouse.

The escalating global challenge of dermatophytoses, compounded by the limitations of conventional antifungal therapies and the emerging threat of resistance, creates a compelling need for novel therapeutic paradigms (Chen et al., 2018; Pereira, 2021; Surur et al., 2024). This study provides the first comprehensive, multi-level investigation into the efficacy and mechanisms of methylene blue-mediated aPDT against *M. canis*, a clinically significant and increasingly prevalent cause of tinea capitis (Zheng et al., 2023). Our findings reveal that the remarkable success of MB-PDT stems from a powerful dual mechanism: first, a direct and potent fungicidal effect, characterized by severe cellular damage consistent with photodynamic-induced oxidative stress; and second, a sophisticated and previously unrecognized immunomodulatory role that restores key parameters of the host’s innate immune system (Figure 11).

Our *in vitro* experiments unequivocally established the potent, light- and concentration-dependent fungicidal activity of MB-PDT against a broad panel of clinical *M. canis* isolates. This efficacy is consistent with the foundational principles of aPDT, which is known to generate reactive oxygen species (ROS) that induce non-specific oxidative damage to multiple cellular targets (Kwiatkowski et al., 2018; Liang et al., 2016). To elucidate the subcellular basis for this potent activity, our TEM analysis provided clear visual evidence of the devastation wrought by MB-PDT. The low MIC values demonstrate potent antifungal activity and an efficacy comparable to that reported for MB-PDT against other key dermatophytes, such as *Trichophyton rubrum* (López-Chicón et al., 2016), suggesting a broad-spectrum potential. The treatment induced a concentration-dependent cascade of damage, with the mitochondria and the cell wall emerging as primary targets. The rapid collapse of mitochondria would trigger an immediate energy crisis and initiate apoptosis (Zhang et al., 2011), while the compromise of the cell wall’s structural integrity would render the fungus osmotically fragile and more susceptible to host defenses (Huang et al., 2010). This multi-targeted assault explains the therapy’s potent efficacy and provides a strong mechanistic rationale for why the development of microbial resistance to aPDT is highly improbable (Surur et al., 2024; Tiwari et al., 2025).

A significant advance of this study was the development and use of a robust murine model that accurately recapitulates the key pathological features of human tinea capitis, including follicular invasion (endothrix) and a supportive inflammatory response (Jin et al., 2009). In this challenging *in vivo* setting, a short course of topical MB-PDT demonstrated unequivocal therapeutic superiority. The rapid clinical resolution and the an 80% mycological cure rate achieved with just two applications stand in stark contrast to the slow and incomplete response observed with topical terbinafine. This finding is of immense clinical importance, as it suggests that a localized, rapidly acting therapy like aPDT could serve as a powerful alternative or adjunct to the long courses of systemic antifungals currently required for tinea capitis (Mayser et al., 2020; Kakourou and Uksal, 2010). Crucially, this potent efficacy is coupled with a favorable safety profile. Our cytotoxicity data align with previous reports that highlight the selective accumulation of cationic photosensitizers like MB in microbial cells over mammalian cells, providing a window of therapeutic selectivity (Morgan et al., 2010).

Perhaps the most significant and novel contribution of this work is the elucidation of the profound immunomodulatory effects of aPDT. Our flow cytometry data revealed that *M. canis* infection induces a specific and dramatic shift in the systemic neutrophil profile, characterized by a marked decrease in the homeostatic Dectin-1^+^/Dectin-2^+^ population and a massive expansion of a Dectin-1^−^/Dectin-2^+^ subset. We propose that this Dectin-1^−^/Dectin-2^+^ phenotype represents a cellular signature of the acute inflammatory response to fungal infection. The dynamic regulation of CLRs like Dectin-1 and Dectin-2 is central to antifungal immunity, orchestrating phagocytosis, cytokine production, and the shaping of adaptive responses (Kerrigan and Brown, 2010; Nikolakopoulou et al., 2020). The downregulation of Dectin-1, the primary receptor for fungal β-glucans (Taylor et al., 2007), could represent a fungal immune evasion strategy or a feature of immature neutrophils mobilized from the bone marrow during acute inflammation (Drifte et al., 2013). Concurrently, the reduced expression of the key antimicrobial enzymes MPO and NOX2 in neutrophils from infected mice suggests a state of functional exhaustion (Wherry and Kurachi, 2015).

Remarkably, treatment with MB-PDT drove a systemic reversal of this pathological immune signature. The therapy not only eliminated the fungal trigger but also actively restored the host’s innate immune system to a state of homeostasis. The restoration of the Dectin-1^+^/Dectin-2^+^ neutrophil population and the significant upregulation of MPO and NOX2 expression indicate that aPDT re-arms the host’s primary antifungal effector cells (Borregaard, 2010). The restoration of MPO is particularly intriguing. MPO is an essential enzyme for the formation of Neutrophil Extracellular Traps (NETs), a critical mechanism for ensnaring and killing large fungal hyphae, which are characteristic of dermatophyte infections (Branzk et al., 2014; Papayannopoulos et al., 2010; Metzler et al., 2011). The restoration of MPO, an enzyme essential for NETosis, provides a strong mechanistic basis for a compelling new hypothesis: aPDT may enhance the clearance of follicular infection by re-arming neutrophils with the ability to form Neutrophil Extracellular Traps (NETs) to ensnare fungal hyphae. While this was not directly measured, it represents a promising avenue for future investigation. This hypothesis could be directly tested via *in vitro* co-culture experiments, where neutrophils from treated and untreated animals are challenged with *M. canis*, followed by visualization and quantification of NETs using DNA-binding dyes like SYTOX Green and immunofluorescence for citrullinated histone H3 (Urban et al., 2009). This immunomodulatory effect is a unique advantage of aPDT.

While this study provides a comprehensive analysis, several limitations should be acknowledged. Our investigation was focused exclusively on *M. canis*. Further studies are needed to determine if these findings extend to other dermatophytes. Moreover, while our immunological analysis of peripheral blood neutrophils provides a valuable window into the systemic host response, likely reflecting changes in hematopoietic output from the bone marrow, we acknowledge that it does not directly characterize the immune infiltrate at the site of infection. Future work employing techniques such as tissue immunofluorescence or flow cytometry of skin digests is warranted to confirm that these beneficial immunomodulatory effects manifest directly within the local tissue microenvironment. Finally, our hypothesis regarding the role of aPDT in promoting NETosis, while mechanistically plausible, remains indirect and warrants direct experimental validation. From a clinical perspective, the rapid efficacy and localized nature of MB-PDT offer a compelling alternative to the lengthy systemic treatments required for tinea capitis, potentially improving patient compliance and reducing systemic side effects. Future investigations could explore the optimization of treatment protocols, such as investigating daylight-activated aPDT to enhance accessibility (García-Malinis et al., 2018), and evaluating its efficacy as an adjunct therapy for severe or refractory cases.

While this study provides a comprehensive analysis, several limitations should be acknowledged. Our investigation was focused exclusively on *M. canis*. Further studies are needed to determine if these findings extend to other dermatophytes. Notably, while our TEM results demonstrate cellular destruction characteristic of oxidative damage, the study was not designed to quantify the generation of reactive oxygen species (ROS). Future work using ROS-specific probes would be required to definitively confirm this fungicidal pathway. Furthermore, our immunological analysis focused on the expression of key enzymes rather than their direct functional activity. Future studies employing functional assays, such as NETosis visualization or oxidative burst measurement, are warranted to determine if the restored expression translates to enhanced functional capacity. Finally, a limitation of our *in vitro* safety assessment is the absence of direct photo toxicity data for the full MB-PDT regimen on host cells, which should be addressed in future work to more precisely define the therapeutic index.

## 5 Conclusion

In conclusion, MB-PDT is a highly effective treatment for *M. canis* infection, with an efficacy derived from a potent dual mechanism: direct fungicidal action and profound modulation of the host innate immune response. This study provides a critical proof-of-concept for this therapeutic approach. While these findings underscore the treatment’s novelty and significance, they also highlight the need for further investigation to fully validate these results. Therefore, despite its high efficacy, favorable safety profile, and low resistance potential making it a robust therapeutic candidate, subsequent studies are necessary before its advancement into clinical trials.

## Data Availability

The raw data supporting the conclusions of this article will be made available by the authors without undue reservation.
